# Postmortem Immunohistochemical Findings in Early Acute Myocardial Infarction: A Systematic Review

**DOI:** 10.3390/ijms25147625

**Published:** 2024-07-11

**Authors:** Oana-Maria Isailă, Oana Mihaela Ion, Robert Luta, Raluca Catinas, Ana Ionita, Diana Haisan, Sorin Hostiuc

**Affiliations:** 1 Faculty of Dental Medicine, Department of Legal Medicine and Bioethics, University of Medicine and Pharmacy ”Carol Davila” Bucharest, 050474 Bucharest, Romania; oana-maria.isaila@umfcd.ro (O.-M.I.); oana.ion@inml-mm.ro (O.M.I.); 2 National Institute of Legal Medicine “Mina Minovici”, 042122 Bucharest, Romania; robert.m.luta@gmail.com (R.L.); raluca.catinas@inml-mm.ro (R.C.); ana.ionita@inml-mm.ro (A.I.); haisandiana@gmail.com (D.H.)

**Keywords:** early acute myocardial infarction, immunohistochemistry, markers

## Abstract

The diagnosis of early acute myocardial infarction is of particular importance in forensic practice considering the frequency of sudden cardiac death and the difficulty of positively identifying it through classical histological methods if survival is less than 6 h. This article aims to analyze potential immunohistochemical markers that could be useful in diagnosing acute myocardial infarction within the first 6 h of its onset. We conducted an extensive evaluation of the literature according to the PRISMA guidelines for reporting systematic literature reviews. We searched the Web of Science and PubMed databases from their inception to 2023 using the following keywords: “myocardial infarction” and “immunohistochemistry”. Fifteen studies met the inclusion criteria. Immunohistochemical markers as complement factors and CD59, myoglobin, fibrinogen, desmin, tumor necrosis factor alpha (TNF-α), P-38, JNK (Jun N Terminal Kinase), transforming growth factor β1 (TGF-β1), cardiac troponins, fibronectin, H-FABP (heart fatty acid binding protein), dityrosine, fibronectin, CD15, IL-1β, IL-6, IL-15, IL-8, MCP-1, ICAM-1, CD18, and tryptase can be used to identify the first six hours of acute myocardial infarction. These markers are mostly studied in experimental animal models. It is necessary to conduct extensive studies on human myocardial tissue fragments, which will involve the analysis of several immunohistochemical markers and careful analysis of the available data on perimortem events, resuscitation, and postmortem intervals in the context of a uniform laboratory methodology.

## 1. Introduction

One of the most frequent causes of sudden cardiac death worldwide is acute myocardial infarction [1,2]. In some cases, given the short survival time after the acute coronary event, there are no obvious changes identifiable either grossly or using classical histopathology methods, which causes uncertainty regarding a positive diagnosis [3]. Nowadays, the postmortem diagnosis of acute myocardial infarction may employ, besides the analysis of gross and microscopic slides (which may allow its detection if the person survives for more 12 h from the onset of the coronary occlusion), various molecular, genetic and biochemical markers, with higher costs and limited availability [4,5]. Recently, minimally invasive or non-invasive postmortem imaging techniques have been shown to be useful to diagnose acute myocardial infarction [6,7]. If death occurs within six hours from the onset of the occlusion, the methods that can be used in forensic practice are more limited, mainly due to their costs. In these cases, the diagnosis of acute myocardial infarction is mostly indirect, undertaken by detecting the narrowing of the coronary lumen without observing myocardial tissue with the morphological appearance of an infarction, which increases the risk of over-diagnosing it and the omission of other potential non-ischemic myocardial pathologies [3,8,9]. Nowadays, a relatively cheap and widely available technique that has been shown to be useful for the diagnosis of acute myocardial infarction is immunohistochemistry, numerous markers being tested, with varying success, for a positive and differential diagnosis.

Although there are useful pre-existing reviews on this topic [10,11,12], the particular aim of our review is to analyze and systematize the immunohistochemical markers presented in the literature concerning effectiveness in the postmortem detection of early acute myocardial infarction, without omitting necessary and useful laboratory elements in this regard, to help focus on a reliable and reproducible range of potential immunohistochemical markers.

## 2. Results

**Figure 1:**
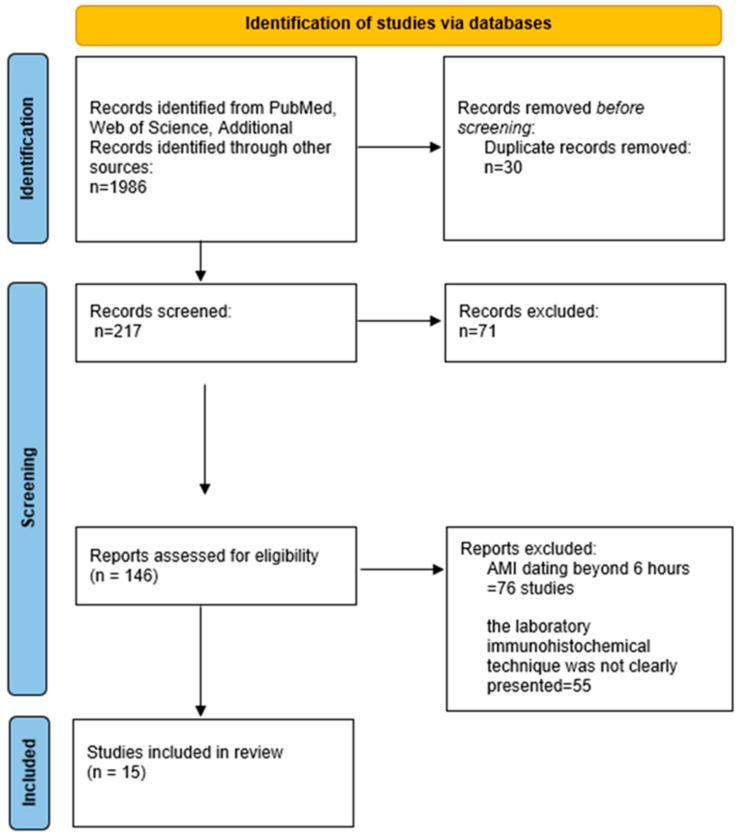
Search synthesis. Prism flow diagram [13].

A number of 1986 studies were initially found. Following their analysis based on the inclusion criteria, we have included 15 articles in this review, as described below (Figure 1, Table 1).

The following IHC markers were discussed in the included studies as potentially effective in dating acute myocardial infarction within 6 h of onset (Table 2).

### 2.1. Complement Factors and CD 59

**Figure 2:**
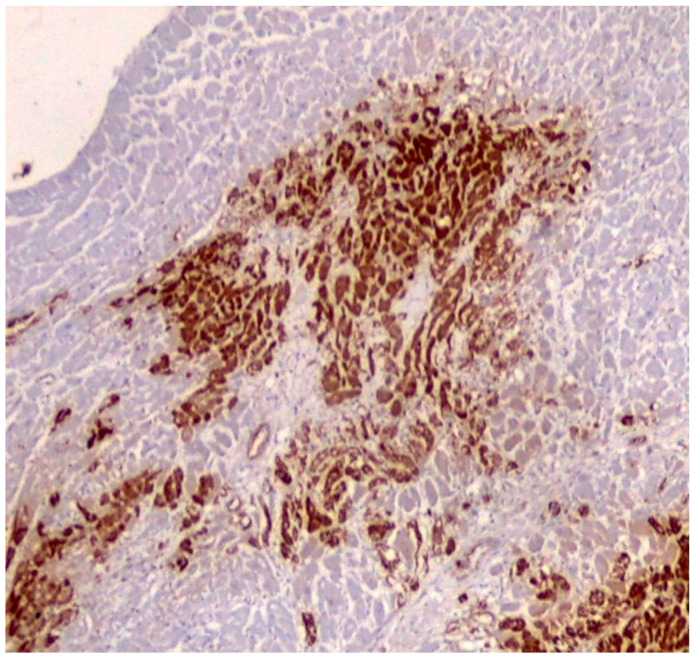
C9-immunostained myocardial necrotic area (4×).

Irreversible ischemic degenerative changes were observed as early as the first 20 min after total coronary obstruction, which allows for the identification of complement complexes at the myocardial level [29]. The membrane attack complex (MAC) of the complement system [30] is part of the C5b-9 membrane complex, an immunohistochemical marker that can be detected in renal [31], muscle [32], and myocardial [33] necrotic biopsy samples. C5b-9 accumulates specifically at the level of necrotic myocardial cells and is associated with cell membrane damage by opening membrane pores [34]. Its concentration depends on the diffusion rate of the complement substrate in the infarcted area, and therefore on reperfusion [35]. Jasra et al., in a human necropsy casework study in which they compared myocardial infarctions more recent than 6 h and myocardial infarctions older than 6 h, concluded that ischemic and infarcted cardiac myofibrils show a positive C9 (Figure 2) staining as early as the first 6 h [25]. CD 59, also called protectin, inhibits the cytolytic activity of the complement system, and therefore myocardial loss and its plasma release occur prior to complement activation [16,36]. From an immunohistochemical standpoint, Väkevä et al. in their studies found that CD59 expression exists at the level of the sarcolemal membrane of the normal myocardium, whereas in the infarcted region after 1–14 days, it is low or absent, accompanied by the concomitant deposition of MAC in CD59-negative areas. Small CD59 vesicles were found at the normal myocardium-infarcted myocardial boundary, suggesting its removal by a possible excretory mechanism [36].

### 2.2. Myoglobin (MB)

Myoglobin is a morpho-functionally comparable protein to hemoglobin; the difference between them lies in the number of polypeptide chains and the number of oxygen-binding sites. Myoglobin has a greater affinity for oxygen compared to hemoglobin and is very effective in extracting oxygen from the blood. It is found in skeletal muscle fiber sarcoplasm and cardiac myocyte cytoplasm [37]. Also, in much lower concentrations, it can be found in smooth muscle, endothelial, and tumor cells, such as in breast, lung, and colon cancer, etc. [38,39], which makes this protein nonspecific. There are studies evaluating the morphological changes and myoglobin content in the normal myocardium versus the ischemic and necrotic myocardium. These studies found a loss of myoglobin from the necrotic myocardium [40]. Considering the criterion for the biohumoral detection of myoglobin level, the concentration peaks 6 h from the moment of coronary obstruction; however, for the microscopic criterion, this time interval is lower, allowing an evaluation of necrotic myocardial tissue [41,42]. Xiaohong et al. found subendocardial cellular myoglobin depletion in mice 30 min after coronary ligation, which expanded transmurally within 3 h [43]. Amin et al., in their necropsy casework study in which they also compared the immunohistochemical expression of MB in cases with definite signs of ischemia, cases with probable signs of ischemia, and cases without signs of ischemia but with other causes of myocardial damage, found variable myoglobin depletion in all cases with definite signs of ischemia and in all cases with other causes of myocardial damage [44].

### 2.3. Fibrinogen

Fibrinogen is a glycoprotein complex that is converted by thrombi to fibrin, and further to a fibrin-based blood clot during the coagulation process. Its increase is known to be a risk factor for AMI and stroke [45], and it was one of the earliest immunohistochemical markers evaluated for the diagnosis of AMI. Raza-Ahmad showed that, in the first 12 h postmortem, a positive reaction for fibrinogen was obtained not only on fibers demonstrating coagulative necrosis, contraction band necrosis, or a wavy pattern, but also in fibers suspected of acute ischemia in the absence of visible changes in H&E [46]. Brinkman et al. performed a case–control study using four groups: AMI macroscopically present, occlusive coronary thrombosis without infarction, coronary atherosclerosis without AMI/occlusion, and a control group. The second group (representing coronary occlusion without gross signs of AMI) is representative of supra-acute MI. In this group, the authors found a patchy and moderate reaction, which was much weaker than the grossly visible AMI group [15].

Xiaohong, in a study using an animal model for early myocardial ischemia, found that, after 30 min, fibrinogen staining was identified as light brown dots in subendocardial myocardial cells. One hour later, the immunostaining was identifiable in the middle myocardium, and after another two hours, in the subepicardial cells. After another three hours, the entire myocardial layer was positive [43]. Sabatasso et al., also on an animal model, found the earliest positive reaction for fibrinogen within an hour of the coronary occlusion, only in the ischemic area, while the non-affected myocardium showed a negative reaction [47].

The intensification of the positive reaction of myocardial fibrinogen together with myoglobin depletion denotes possible irreversible myocardial lesions detectable from the first 30 min of the moment of coronary blood flow arrest [15]. However, the reliability of fibrinogen is considered to be low given the potential false-positive results from contaminated samples [43].

### 2.4. Desmin

Desmin is a protein specific to muscle tissue; it is structural to the heart muscle but also skeletal and smooth muscles. It plays an essential role in maintaining the structural and mechanical integrity of the contractile structure. For example, in rat studies, a deficiency in desmin synthesis was found to cause dilated cardiomyopathy, smooth muscle defects, and skeletal myopathy [48,49]. In myocardial ischemia, the depletion of desmin from the cytoplasm of the heart cell begins 30 min after the onset of ischemia, and within 90–120 min, depletion is complete [50].

### 2.5. Tumor Necrosis Factor Alpha (TNF-α), P-38, and JNK (Jun N Terminal Kinase)

TNF-α is a cytokine secreted by macrophages that can be detected mainly in vascular smooth muscle cells. Increased values have been reported in heart failure [51], but also in infarcted myocardial tissue, with TNF-α having the role of stimulating the proliferation and expression of fibronectin at the level of fibroblasts in the injured myocardium [52].

P-38-activated kinase (MPAK) is interconnected with cardiac activity; its isoforms have a role in the growth and differentiation of cardiomyocytes [53]. It is also closely related to oxidative stress, along with nuclear factor kappa B (NF-k-B), which plays a role in the production of TNF-α [54] and JNK (stress-signaling kinases) [55].

### 2.6. Transforming Growth Factor β1 (TGF-β1)

TGF-β1 is a profibrotic cytokine found in increased amounts in acute myocardial infarction. It also precedes increased collagen expression [56].

### 2.7. Cardiac Troponins

cTnT, a marker of myocardial lesions, is a component of the troponin complex of the muscle cell. Serum levels of cTnT increase within 4–6 h of the onset of acute myocardial infarction and peak at about 24 h [57]. Fishbein et. al, in an experimental immunohistochemical study in animals, found an uneven loss of cTnT alongside cTnI in the periphery of the necrotic myocardium, with the loss of cTnT being more pronounced compared to that of cTnI. In some cases, histopathological changes are detectable as early as the first 30 min after coronary occlusion [58]. In the study by Amin et al. on human subjects, the results were similar [44]. Diaz et al., in their necropsy casework study, found in cardiac deaths a more diffuse and pronounced immunohistochemical expression of cTnC compared to that of cTnT within the first hour after the onset of acute myocardial infarction [22].

### 2.8. H-FABP (Heart Fatty Acid Binding Protein)

H-FABP (heart fatty acid binding protein) is a cardiac protein that plays an essential role in the metabolism of cardiomyocyte fatty acids [59]. At the time of ischemic myocardial injury, H-FABP is released into the blood and eliminated renally. Biohumorally, it can be detected from about an hour from the onset of myocardial infarction [60], and immunohistopathologically it is considered objectifiable in the first 4 h after blocking the coronary flow.

### 2.9. Dityrosine

Dityrosine is a stable product synthesized by the myeloperoxidase–hydrogen peroxidase system of neutrophils and macrophages. This is a marker of oxidative protein stress [61]. In the study on human necropsy cases conducted by Mayer et al., it was found that dityrosine, although not specific to infarcts, can also be a marker of myocardial infarction with a short survival time, being detectable immunohistochemically after the first 4 h of survival after the onset of infarction [27].

### 2.10. Fibronectin

**Figure 3:**
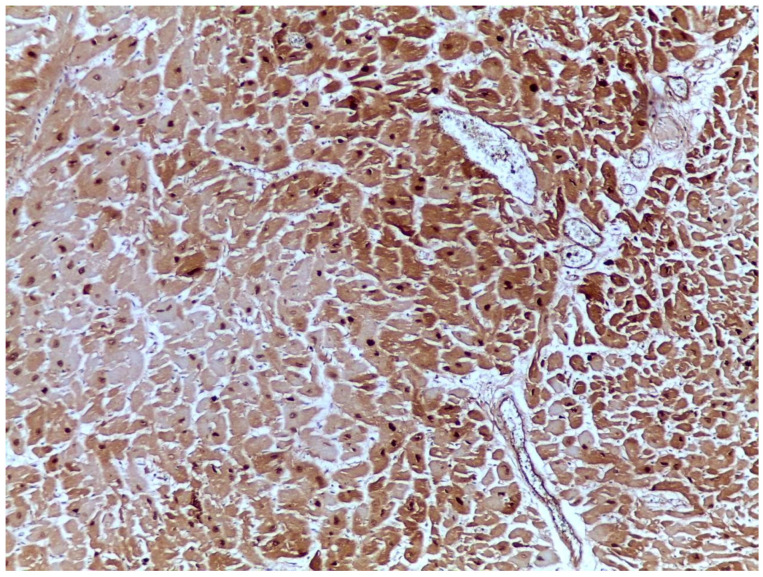
Fibronectin-immunostained myocardial necrotic area (10×).

Fibronectin (Figure 3) is an extracellular matrix protein assembled into viscoelastic fibrils that can bind growth factors and cytokines. These fibrils play an essential role in injury healing processes, possessing unique mechanical properties that allow them to modify detected mechanotransduction signals transmitted by cells [62,63,64]. In the experimental study conducted by Casscells et al. to observe the immunohistochemical aspects of fibronectin in acute myocardial infarction, it was found that fibronectin was irregularly located in the cytoplasm and in the interstitial space of myocytes of the area irrigated by the ligated coronary infarction [14]. Saleki et al. performed a semiquantitative analysis of fibronectin immunostaining in three study groups—confirmed AMI, suspected AMI, and negative controls—and employed a four-stage grading for each case (negative to 3+). From 15 cases of confirmed AMI, a strong immunostaining pattern was identified in 10, both in the cytoplasm and nuclei of the affected fibers, in 4—a moderate pattern, and in 1—a weak staining. In the negative controls, 13 had a negative reaction, 4 had a weak reaction, and 1 a moderate reaction [65]. This suggests that the sensibility of a strong immunostaining pattern is high for AMI, but moderate and weak patterns may be encountered in other pathologies as well, possibly caused by terminal ischemia associated with an increased agonal period. Hu et al. tried to evaluate the specificity of positive fibronectin immunostaining for the detection of AMI by quantifying the total positive area using the Interactive Building Analysis System. They found that, in AMI, the positive staining area was around 100 times higher compared to normal controls, hemorrhagic shock, cardiac contusion, and organophosphate poisoning, around 75 times higher compared to mechanical asphyxia and electrocution, and only around 2.5 times higher compared to myocarditis. Their study of fibronectin in necropsy cases additionally found that it is not affected by postmortem autolysis nor by prior formalin fixation, which also allows for the retrospective analysis of cases of forensic interest to detect early myocardial infarction postmortem [66].

### 2.11. CD 15

CD15, or Lewis x antigen, is a cell-surface glycan, considered a useful marker in identifying cells of the granulocyte series [67]. Given the hypothesis that from the first hours from the onset of acute myocardial infarction, leukocytes are gradually attracted to the infarcted myocardial area, some studies have tried to estimate the age of acute myocardial infarction based on the number of CD15-positive marked cells [65,68]. The number of CD-15 positive cells is one of the reliable immunohistochemical markers in dating myocardial infarction, as a linear relationship with time has been found regarding the accumulation of polymorphonuclear leukocytes at this level [69]. For example, in the study conducted by Mortensen et al., it was estimated that several myocardial-positive CD15 cells between 20 and 30 raised the suspicion of an acute myocardial infarction, and values above 30 were considered relevant for an acute myocardial infarction occurring for 5–6 h [68].

### 2.12. CD 18

CD 18 is a cell-surface adhesion molecule that shows increased expression on the cell membrane in the infarcted area. It is associated with neutrophil activation. CD18 leukocyte integrins are adhesion receptors released in the acute phase and easily measured. They are mainly involved in neutrophil extravasation [70,71]. Hill et al., in their experimental rabbit heart study, found an increase in CD18 expression as early as the first 20 min after infarction and correlated it with the extent of myocardial necrosis, observing, however, an interindividual variation that did not allow for percentage/numerical appreciations [71].

### 2.13. ICAM 1

ICAM 1 is an endothelial receptor and ligand for leukocyte integrin CD 18. It allows their firm adhesion to the vascular endothelium [72]. There are studies that have demonstrated the increase in soluble ICAM 1 in the context of acute myocardial infarction [73], but also its overregulation in human cardiomyocytes after infarction. However, this is not detectable in infarcts more recent than one day [74].

### 2.14. ILs (Interleukins)

ILs are important inflammatory mediators of acute myocardial infarction, with a role in exacerbating (e.g., IL6) or attenuating (e.g., IL10, IL 15) the acute inflammatory response. They are released from various cells into the bloodstream and interstitium, where they bind to interleukin receptors on cell surfaces, causing cell activation. Some studies have also analyzed the prognosis of acute myocardial infarction according to the level of serum interleukins [75,76].

### 2.15. Monocyte Chemoattractant Protein-1 (MCP 1)

MCP 1 is a chemokine that induces the recruitment and activation of monocytes, T cells, and NK cells [77]. It is produced by many cells in response to injury factors or exposure to other cytokines, and is also involved in acute myocardial infarction [78]. The antiapoptotic and chemotactic effects of MCP 1 may be mediated by different signaling pathways in the infarcted myocardial area [79]. In the study conducted by Turillazzi et al., a strongly expressed response of MCP-1 was found in the early phase of acute myocardial infarction in the first 0–4 h [26].

### 2.16. Tryptase

Tryptase is a protease released by mast cells. The increase in mast cell tryptase plays a central role in the immediate inflammatory and allergic reactions initiated by IgE [80]. It induces fibroblast proliferation, stimulates fibroblast chemotaxis, and regulates the increased production of collagen type I [81,82]. The same study mentioned above by Turillazzi et al. found a moderate immunohistochemical reaction for tryptase at myocardial infarction sites 0–6 h old, along with CD15, IL-1β, IL-6, TNF-α, IL-8, CD18, ICAM-1, IL-15, and MCP-1 [26].

### 2.17. Adiponectin

Adiponectin is an adipokine secreted by adipocytes, with anti-inflammatory, antifibrotic, antioxidant, and cardioprotective effects [83,84]. Experimental studies on animals have proved that in ischemic myocardial lesions, adiponectin accumulates in the heart tissue, where it passes from the vascular compartment. It has a longer half-life in heart tissue than in plasma [85].

### 2.18. Macrophage Migration Inhibitory Factor (MIF)

MIF is an immunoregulatory cytokine considered a potential biomarker in numerous diseases that also have an inflammatory component [86,87]. It can be produced by monocytes, macrophages, endocrine, epithelial, and endothelial cells, is stored cytoplasmically, and is rapidly released to stimuli such as microbial products, proliferative signals, and hypoxia [88,89,90]. In the case of acute myocardial infarction, MIF increased in myocytes within a few hours, preceding macrophage infiltration in the infarcted area [20].

## 3. Discussion

Our review has exposed the main immunohistochemical markers considered over time for diagnosing myocardial infarction more recent than 6 h. They derive from knowledge of the pathophysiological mechanism, and appropriate laboratory elements are required for their determination, as well as complex and expensive forensic analysis [11]. As far as laboratory methods are concerned, there is a need to simultaneously analyze as many markers of recent acute myocardial infarction as possible on the same tissue sample [91]. However, particular aspects of each case must also be considered, such as cardiopulmonary resuscitation, catecholamine injection, drug use (ecstasy, cocaine), agonal artifacts, postmortem interval, autolysis, and pre-existing ischemic events, which can influence immunohistochemical results [47,92].

### 3.1. Agonal Myocardial Immunohistochemical Changes

Myocardial contraction bands can be observed in multiple circumstances of death, as a result of artefactual causes such as poor sampling techniques (e.g., a low-temperature fixator). In experimental studies on ligated pig hearts, bands were found with abundance in the first 20–30 min [93]. In the study conducted by Morita et al. on necropsy fragments of human heart, in order to differentiate pathological contraction bands from artefactual ones by immunohistochemical analysis, CCC9-positive reactions (complement C9 fraction) and SIRT 1-negative reactions were found in cases of myocarditis and myocardial ischemia, and in the rest of the cases, who benefited from cardiopulmonary resuscitation but had various causes of death, a positive reaction was found only in the case of SIRT 1, concluding that the positive CCC9 marker allows for the differentiation between myocardial changes in acute myocardial infarction and myocardial changes occurring after attempts at cardiopulmonary resuscitation or other terminal conditions [94].

Depending on the immunohistochemical reaction obtained, they proposed the following classification of contraction bands (Table 3).

In addition, CCC9 is detectable from the early stage of acute myocardial infarction. Classical hematoxylin–eosin staining does not allow the detection of changes in this regard.

Therefore, a myocardial-positive CCC9 immunohistochemical reaction is a reliable element supporting early acute myocardial infarction [95], while also allowing the differential diagnosis of agonal changes [94]. Sabatosso et al., in their comparative immunohistochemical study of cardiac changes in myocardial ischemia, acute myocardial infarction, and hanging, using necropsy casework, found that myoglobin and TnT showed no expressions, supporting the statistically significant differences between the three groups. This, for example purposes, makes the specificity of immunohistochemical markers in humans inconclusive. Moreover, in the same study, a greater positive expression of Cx34 and Jun B markers was observed in cases of hanging than in cases of acute myocardial infarction or myocardial ischemia, demonstrating their non-specificity in humans, unlike experimental animal studies [96].

### 3.2. Myocardial Immunohistochemical Changes Following Autolysis and Putrefaction

In general, immunohistochemical results can also be influenced by biological changes during a prolonged postmortem interval with subsequent autolysis and putrefactive phenomena, which, against the background of acidosis, determines the activation of intracellular enzymes with the degradation of protein structures [12,97].

In this regard, the study by Thomsen and Held demonstrated that the marker C5b-9 can be detected in the myocardium at a postmortem interval of up to 11 days [98]. Ortmann et al. also obtained a positive reaction for C5b-9 at postmortem intervals of about 8 weeks [99]. Fibronectin and myoglobin could be detected immunohistologically at approximately 3 days postmortem at the myocardial level [15,42]. However, these studies were not case-controlled and were performed on a small number of subjects, which may make it difficult to differentiate between false-positive results, against the background of protein degradation by autolysis and putrefaction, and positive results in a real pathological context following acute myocardial infarction [12]. Hu et al., in their study in which they analyzed the expression of desmin, actin, and myoglobin on heart fragments, stored at 4C and analyzed at different postmortem intervals, found that the postmortem interval had a significant influence on the experience of the markers studied, with the markers being resistant to autolysis only for an interval of 2 days postmortem [97].

### 3.3. Perimortem Catecholamines

High levels of catecholamines (either exogenous or endogenous, e.g., in pheochromocytoma or brainstem lesions affecting the nucleus tractus solitari [100]) have myotoxic action. In experimental animal studies, immunohistochemical myocytic apoptotic phenomena were observed 3–6 h after catecholamine injection, and necrotic phenomena were observed at about 18 h. In the experimental study on mice conducted by Goldspink et al., these phenomena were detected as heterogeneous myocardial, and were more pronounced in the left ventricular subendocardial with the predominance of necrosis phenomena and positive caspase 3 and myosin reactions [101]. Another experimental study on rats by Lu et al. noted that an overdose of norepinephrine (NE) can cause severe cardiopulmonary dysfunction, with cardiac immunohistochemical positivity within the first 6 h of TnT and Cx43 at the biventricular and septal levels [102].

### 3.4. Gender and Myocardial Ischemic Preconditions

A person’s gender and pre-existing ischemic conditions impact the severity and course of acute myocardial infarction. Some studies have concluded that the severity of apoptosis and myocardial cell necrosis is more pronounced in men [103]. Even experimental studies in mice have detected smaller areas of acute myocardial infarction in females than in males [104]. Pre-existing ischemic conditions refer to transient myocardial ischemic episodes in the subject’s pathological history and have been found to have a protective role for subsequent cardiac ischemic injury, resulting in infarction in a smaller area and a better preservation of left ventricular function [105,106]. In the experimental case–control study conducted by Scholl et al. in mice, in which they analyzed the expression of dityrosine, TnT, TnI, and Connexin 43 (Cx43) according to the sex of the subjects and pre-existing myocardial ischemic conditions, it was concluded that only pre-existing ischemic conditions had a significant impact on the expression of troponins and Cx43, with sex having no discriminatory influence on the results obtained [107].

### 3.5. Overall Considerations

Overall, the results of this review find their usefulness in forensic practice, systematizing the main immunohistochemical markers to be analyzed in the case of suspicion of an early acute myocardial infarction. After the first 6 h, these markers can be detectable for certain time intervals (see Table 1—Included studies) with the coexistence of the usual morphological changes in acute myocardial infarction, detectable by classical optical microscopy, which can help better characterize acute myocardial infarction. Many markers show significant changes dependent on the time interval, as has been extensively evaluated elsewhere [10,11,12] in extensive, comparative analyses. The studies existing so far in the analyzed databases are mainly experimental and performed on non-human subjects, but have an important contribution to forensic practice in the instance of the suspicion of early acute myocardial infarction, undetected by classical microscopy techniques. In future, the simultaneous analysis of a panel of markers will be necessary to increase diagnostic accuracy, requiring appropriate laboratory infrastructure and reagents [56]. Also, extensive comparative studies are needed to analyze the specificity of the presented immunohistochemical markers.

The limitations of this review are represented by the absence of the specificity of these markers for early acute myocardial infarction, being rather markers of acute myocyte necrosis in general, regardless of etiology, and the small number of studies aimed at the immunohistochemical analysis of acute myocardial infarction more recent than 6 h. Another limit is represented by interspecies variability in marker analysis, with most markers being analyzed on non-human subjects. In experimental studies, human tissue fragments may show limited specificity. Consequently, this requires the survey of a larger number of tissue samples, the simultaneous analysis of several immunohistochemical markers, and the careful analysis of survey data regarding perimortem events, resuscitation, and postmortem intervals.

## 4. Materials and Methods

We conducted a study in adherence to the PRISMA guidelines for reporting systematic literature reviews. In this regard, we searched the Web of Science and PubMed databases from inception to 2023, using the keywords “myocardial infarction” and “immunohistochemistry”. The inclusion criteria were observational studies that analyzed the effectiveness of immunohistochemical markers in the postmortem diagnosis of early acute myocardial infarction (the first 6 h after the occurrence) and exposed the laboratory techniques and materials used. We excluded reviews and meta-analyses. We also analyzed the reference lists of the studies included from the 2 databases to add potential supplementary studies to this literature review. The main analyzed elements in each study were as follows: the number of subjects, type of subjects (humans/non-humans), age of the subjects, documented pathologies of the subjects, symptom–death time interval, laboratory equipment and laboratory techniques used (see Supplementary Table S1—Laboratory methodology in the included studies), and immunohistochemical marker(s) analyzed. The review is registered in the Open Science Framework: https://doi.org/10.17605/OSF.IO/2GWUT (accessed on 8 July 2024).

## 5. Conclusions

Sudden cardiac death still poses a challenge to necropsy practice. Although the pathophysiological mechanisms of acute myocardial infarction have been carefully analyzed and unraveled through studies conducted on both human and animal subjects, we have a reduced range of feasible immunohistochemical markers useful in diagnosing early acute myocardial infarction in human subjects. Extensive studies on human necrotic tissue fragments are required with the simultaneous analysis of several immunohistochemical markers, including survey data for perimortem events, resuscitation, and postmortem intervals in the context of a uniform laboratory methodology.

## Figures and Tables

**Table 1:** Included studies.

Authors_Year	Dating Time Frame of Myocardial Infarction	Subjects	IHC Markers
Casscells et al._1990 [14]	4, 24, 48, 72 h, and 7 days	Sprague Dawley rats	Fibronectin
Brinkmann et al._1993 [15]	0–80 h	Humans	Myoglobin, desmin, fibrinogen, complement C5b-9
Vakeva et al._1994 [16]	1, 2, 3, 6, 24, or 72 h	Wistar rats	Complement C 1,3,8,9; CD59
Mathey et al._1994 [17]	15–30 min, 1 h, 1.5 h, 2 h, 3 h, 5–6 h, 12–17 h, 22–29 h	Rabbits	C5b-9
Robert-Offerman et al._2000 [18]	<6 h, 6–24 h, 24–72 h, day 4–8, >8 days	Humans	Complement factor C9, membrane attack complex (MAC) of complement
Dai et al._2002 [19]	1 h, 6 h, 24 h, 3 days, and 7 days	Wistar rats	TNF-α, TGF-β1
Yu et al._2003 [20]	6 h, 1 day, 3 days, 1 week, or 2 weeks	Male Sprague Dawley rats	Macrophage migration inhibitory factor (MIF)
Sumitra et al._2005 [21]	1st, 2nd, 4th, 8th, 16th and 32nd hour	Wistar rats	C5, C6,C7, C8, C5b-9
Díaz et al._2005 [22]	2–16 h postmortem	Humans	Cardiac troponin C (cTnC) and cardiac troponin T (cTnT)
Meng et al._2006 [23]	15 min, 30 min, 1 h, 2 h, 4 h, 8 h	Humans, Wistar rats	Heart fatty acid binding protein (H-FABP)
Dai et al._2007 [24]	1 h, 6 h, 1 day, 3 days	Wistar rats	tumor necrosis-alfa, p38, JNK
Jasra et al._2012 [25]	<6 h, >6 h	Humans	Cardiac troponin-I (CT-I) and complement C9 (C9).
Turillazzi et al._2014 [26]	From 0–6 h to more than 12 h	Humans	CD15, IL-1β, IL-6, TNF-α, IL-15, IL-8, MCP-1, ICAM-1, CD18, tryptase
Mayer et al._2014 [27]	4–24 h	Humans	Dityrosine, fibronectin, C5b-9
Gozalo et al._2022 [28]	0–30 min–2 months	Owl monkeys	C9 complement

**Table 2:** Immunohistochemical markers systematized according to the time interval specified in the studies included in the analysis.

Timeframe	IHC Markers
1 h	C9 complement, myoglobin, desmin, fibrinogen, C5b-9, tumor necrosis alpha, p-38, JNK, TGF-β1, cTnT, CD59.
2 h	cardiac troponin C (cTnC)
4 h	H-FABP (heart fatty acid binding protein), dityrosine, fibronectin, CD15, IL-1β, IL-6, IL-15, IL-8, MCP-1, ICAM-1, CD18, tryptase
6 h	MIF

**Table 3:** Classification of contraction bands according to CCC9 and SIRT 1 immunohistochemical reactions, according to Morita et al.

Contraction Bands	Particularities
Type 1	Induced by high fever exposure
Type 2	CCC9-positive reaction Specific to acute myocardial infarction
Type 3	CCC9-negative reaction, clear SIRT 1-negative reaction Induced by exposure to low temperatures
Type 4	CCC9-negative reaction, SIRT 1-positive or inconclusive reaction Induced by cardiopulmonary resuscitation attempts

## Data Availability

PubMed and Web of Science databases.

## Supplementary Materials

The following supporting information can be downloaded at: https://www.mdpi.com/article/10.3390/ijms25147625/s1.
