# Supplementary material for: Postmortem Immunohistochemical Findings in Early Acute Myocardial Infarction: A Systematic Review
Source: Int J Mol Sci. 2024 Jul 11;25(14):7625. doi: 10.3390/ijms25147625 (PMC11277133; doi:10.3390/ijms25147625)
Supplement: Supplementary file 1 [file ijms-25-07625-s001.zip › ijms-3043833-supplementary.pdf]

Table S1. Laboratory Methodology in the included studies (Supplementary file)

**Authors_year**	**IHC markers**	**Laboratory method**
Gonzalo et al._2022	C9 complement	„paraffin-embedded tissues cut to a thickness of 5μm, placed on Probe-On Plus slides (Fisher Scientific, Pittsburgh, PA). The tissue was rehydrated, antigen retrieved with citrate buffer, pH 6.0 for 20 mins in a pressure cooker and allowed to cool to room temperature (RT). The slides were subsequently rinsed with TBST except dH2O was used for the final rinse. Slides were incubated with SNIPER (Biocare Medical, Pacheco, CA) for 15 mins and rinsed with TBS. Blocked with Peroxidazed (Biocare Medical, Pacheco, CA) for 5 minutes. Slides were incubated with rabbit antihuman complement C9 polyclonal antibody for 45 minutes (1:50, MyBioSource, San Diego, CA, catalog # MBS2401555) followed by two TBS rinses. Mach 2 (Biocare Medical, Pacheco, CA) was applied for 25 minutes and DAB+ (Biocare Medical, Pacheco, CA) was applied for 4 minutes. All slides were counter stained with CAT Hematoxylin (1:5) for two minutes."[28]
Dai et al._2007	tumor necrosis-alfa, p38, JNK	„Tissue samples were perfused with 2% paraformaldehyde for cryosection. Tissue samples were first immersed in 4% normal goat serum (Vector Laboratories, Burlingame, CA) for 1 h to block non-specific binding. They were then incubated overnight at room temperature (25 °C) in rabbit anti-TNF-a (dilution 1 : 200, Chemicon), rabbit anti-phospho-p38 (dilution 1 : 200, Cell Signaling) or rabbit anti-phospho-JNK (dilution 1 : 200, Cell Signaling). The secondary reagents used were biotinylated goat anti-rabbit IgG and ABC kit (Vector Laboratories). The peroxidase reaction was visualized using 3,3' -diaminobenzidine tetrahydrochloride (DAB, Sigma Chemical Co.) as a peroxidase substrate."[24]
Yu et al._2003	Macrophage migration inhibitory factor (MIF)	„Double immunostaining of MIF expression by infiltrating macrophages in infarcted or non-infarcted heart tissues was performed on 4-m paraffin sections of formalin-fixed heart with a microwave-based, multiple immunoenzymatic stainin using monoclonal anti-MIF (III.D.9 IgG) and mouse monoclonal anti-ED-1 antibodies"[20]
Dai et al._2002	TNF-α, TGF -β1	„The hearts were fixed for 24 h with 4% paraformaldehyde and cryoprotected by immersion in 20% sucrose in phosphate buffer. Transverse sections of the peri-sinoatrial nodal area were cut at a thickness of 20 μm on a cryostat and mounted on 3-aminopropyltriethoxy-silane-coated slides. The primary antisera used were goat anti-TNF-α (R

		and D systems), and goat anti-TGF-β1 (Santa Cruz Biotechnology). The secondary reagents used for cytokine localizations were biotinylated rabbit anti-goat IgG and ABC kit (Vector Laboratories). The peroxidase reaction was visualized by using 3,3'-diaminobenzidine tetrahydrochloride (Sigma) as a peroxidase substrate."[19]
Mayer et al._2014	dityrosine, fibronectin, C5b-9	„The samples were fixed in formalin and embedded in paraffin. IHC -Tissue sections were deparaffinised and washed in distilled water three times for 5 min, then washed twice in Tris-buffered saline (TBS)-buffer with 0.5 % Tween 20 for 5 min.<br>The primary antibody against dityrosine (Acris, mouse, AM 20243PU-S) was used in a concentration of 1:600, slides were incubated overnight at +4 °C. Slides were then washed in TBS-buffer with 0.5 % Tween 20 twice for 5 min. Endogenous peroxidase was blocked with 0.03 % H2O2 for 15 min; afterwards, slides were washed in distilled water twice for 5 min and in TBS-buffer with 0.5 % Tween 20 twice for 5 min. Then slides were incubated with a peroxidase-marked polymer (Medac, Histofine® Simple Stain MAX PO against mouse, 413132) for 30 min.<br>For fibronectin slides were treated with proteinase K (Dako, S3020) for 7 min and washed twice in TBS-buffer with 0.5 % Tween 20 for 5 min. The primary antibody against fibronectin (Biozol, rabbit, DAK-A024502-2) was used in a concentration of 1:2,000, slides were incubated overnight at +4 °C. Slides were then washed in TBS-buffer with 0.5 % Tween 20 twice for 5 min. Endogenous peroxidase was blocked with 0.03 % H2O2 for 5 min. Afterwards, slides were washed in distilled water twice for 5 min and in TBS-buffer with 0.5 % Tween 20 twice for 5 min before being incubated with a peroxidase-marked polymer (Medac, Histofine® Simple Stain MAX PO against rabbit, 414142) for 30 min.<br>The primary antibody against C5b-9 (Abcam, rabbit, ab 55811) was used in a concentration of 1:150, slides were incubated overnight at +4 °C. Slides were then washed in TBS-buffer with 0.5 % Tween 20 twice for 5 min. Endogenous peroxidase was blocked with 0.03 % H2O2 for 30 min; afterwards, slides were washed in distilled water twice for 5 min and in TBS-buffer with 0.5 % Tween 20 twice for 5 min. Then slides were incubated with a peroxidase-marked polymer (medac, Histofine® Simple Stain MAX PO against rabbit, 414142) for 30 min.<br>Tissue samples were washed in TBS-buffer with 0.5 % Tween 20 twice for 5 min and were then stained with substrate-chromogen AEC (MEDAC, AEC + (3-amino-9-ethylcarbazole) Substrate-Chromogen, K3469). Afterwards, the slides were washed with distilled water for 5 min, counterstained with Mayers Hematoxylin (Merck, Mayers hemalum solution, 1092492500) and washed again in

		distilled water and tap water. Sections were mounted with Aquatex® (Merck, 1.05862.0050)."[27]
Brinkmann et al._1993	myoglobin, desmin, fibrinogen, complement C5b-9	„Using indirect immunohistochemistry (IH) the following antigens were investigated: Primary antibody. Polyclonal rabbit; anti human antibodies against desmin, myoglobin, fibrinogen; dilution 1/50; Dako, D22047 Hamburg. For C5b-9 complement-complex (C5b-9): anti-human C5b-9; polyclonal rabbit; dilution 1/10; Calbiochem, D-65812 Frankfurt/M. Secondary antibody. For myoglobin and fibrinogen: swine immunoglobulin to rabbit immunoglobulin; dilution 1/50; Dako, D22047 Hamburg. For desmin: biotinylated rabbit immunoglobulin to sheep/mouse immunoglobulin; dilution 1/500, Boehringer, D-6800 Mannbeim. For C5b-9 comp.: biotinylated rabbit immunoglobulin to mouse immunoglobulin; dilution 1/300, Dako, D-22047 Hamburg. Third step. For myoglobin and fibrinogen: PAP-complex, Dako, D-22047 Hamburg. For desmin: Streptavidin-complex, Boehringer, D-6800 Mannheim. For C5b-9 comp.: Avidin-Biotin-complex, Dako, D-22047 Hamburg. Substrates. Myoglobin, fibrinogen and C5b-9 comp.: 3-Amino-9-ethylcarbazole (AEC), Sigma, D-8024 Deisenhofen. Desmin: 3,3-Diaminobenzidine-tetrahydrochloride (DAB), Amersham, United Kingdom. We used the following structures as positive controls: normal human myocardial fibres for desmin and myoglobin; for fibrinogen its presence in vital haemorrhages (Berg 1975); for C5b-9 comp. its presence in vital muscle lesions (Fechner et al. 1993). Structures that are known to lack the relevant antigens served as negative controls. Furthermore, in each experiment one reagent was omitted from the necessary components, e.g. the primary antibodies. The strength of the reactions and/or their extent were scored semiquantitatively using a 4-degree scale: 0 = negative, 1 = slight, 2 = moderate, 3 = extensive."[15]
Ward Casscells et al._1990	Fibronectin	„For immunohistochemical staining the sections were deparaffinized and rehydrated. Endogenous peroxidase activity was quenched by treatment with 0.6% hydrogen peroxide in methanol for 30 minutes at room temperature. The sections then were washed with 0.25% Brij 35 (Sigma Chemical Co., St. Louis, MO) in 0.01 mol/l TRIS-HCIbuffered saline, pH 7.4 (washing buffer), at room temperature. Then sections were incubated in 0.4% pepsin (P-7125, Sigma) in 0.01 N HCI at 370C for 15 minutes, and rinsed with washing buffer."[14]
Robert-Offerman et al._2000	complement factor C9, membrane attack complex (MAC) of complement	„Serial 4 µm sections were cut from the paraffinembedded blocks and allowed to dry overnight at 37 °C. After deparaffinization and rehydration, the sections were immersed in 1% methanol/H2O2 for 15 min and subsequently rinsed in phosphate buffered saline (PBS).

		The sections were preincubated for 10 min with \`Antibodydiluent' (DAKO, USA) for non-specific staining, which was also used for antibody dilution during the entire staining protocol. Excessive fluid was removed from the slides and sheep antihuman complement factor C9 (1 : 600; The Binding Site, Birmingham, UK) was added; the sections were incubated for 45 min at room temperature (RT). After rinsing with PBS, the sections were incubated for 45 min with biotinylated donkey anti-sheep antibody (1 : 100; The Binding Site, Birmingham, UK) plus 100 ml/ml normal human serum (NHS) (CLB, Amsterdam, The Netherlands), also at RT. Staining was visualized by streptavidin peroxidase and amino-ethylcarbazole (AEC) (Sigma, St. Louis, MO, USA). Subsequently, the sections were counterstained with haematoxylin and mounted for light microscopic evaluation. Parallel sections were routinely H&Estained. As a negative control, keratin-7 as control antibody and omission of the primary antibody were used in order to preclude non-specific binding of the primary and secondary antibodies to human myocardial tissues."[18]
Turillazzi et al._2014	CD15, IL-1β, IL-6, TNF-α, IL-15, IL-8, MCP-1, ICAM-1, CD18, tryptase	„An immunohistochemical investigation of all the samples was performed utilizing a panel of antibodies (CD15, DAKO, Copenhagen, Denmark), IL-1β (Santa Cruz, CA, USA), IL-6 (Santa Cruz, CA, USA), TNF-α (Santa Cruz, CA, USA), IL-15 (R&D Systems, Minneapolis, MN, USA), IL-8 (Abcam, Cambridge, UK), MCP-1 (Santa Cruz, CA, USA), ICAM-1 (Santa Cruz, CA, USA), CD18 (Abcam, Cambridge, UK), tryptase (Novus Biologicals, Littleton, CO, USA). To obtain a better definition of early infarction, we matched the samples with very early markers of necrosis such as cellular antigen troponin C (Novocastra Leica Biosystems GmbH, Nussloch, Germany) and Troponin I (Thermo Fisher Scientific, Fremont, CA, USA). We used 4 mm-thick paraffin sections mounted on slides covered with 3-amminopropyl-triethoxysilane (Fluka, Buchs, Switzerland). Pre-treatment was necessary to facilitate antigen retrieval and to increase membrane permeability to antibodies antiCD 15, IL-1β, MCP-1, IL-15, ICAM-1, CD 18, IL-8, troponin C and troponin I boiling in 0.25 M EDTA buffer, to antibodies anti-TNF-α boiling in 0.1 M Citric Acid buffer, to antibody anti- IL-6 and tryptase for 15 min. in Proteolytic Enzyme (Dako, Copenhagen, Denmark), at 20°C. The primary antibody was applied in a 1:50 ratio CD 15, in a 1:4000 ratio IL-1β, in a 1:2000 ratio IL-6, in a 1:600 ratio TNF-α, in a 1:100 ratio IL-15, in a 1:500 ratio IL-8, ICAM-1 and MCP-1, in a 1:1000 ratio tryptase, in a 1:200 ratio CD 18, in a 1:3000 ratio troponin I and in a 1:6000 ratio troponin C and incubated for 120 min at 20°C. The detection system utilized was the LSAB + kit (Dako, Copenhagen, Denmark), a refined avidin–biotin technique

		in which a biotinylated secondary antibody reacts with several peroxidase conjugated streptavidin molecules. The positive reaction was visualized by 3.3-diaminobenzidine (DAB) peroxidation, according to standard methods. The sections were counterstained with Mayer's haematoxylin, dehydrated, coverslipped and observed in a Leica DM4000B optical microscope (Leica, Cambridge, UK). A semi-quantitative evaluation of the immunohistochemical findings was made by two different investigators without prior knowledge. The reactions were graded as follows: 1. (0): not expressed, 2. (+): isolated and disseminated expression, 3. (++): expression in widespread foci, 4. (+++): widespread expression. All measurements were carried out using the same magnification of image (10×) and by the same two examiners. A third blind microscopic evaluator was involved to weigh the histological evidence. The samples were also examined under a confocal microscope, and a three-dimensional reconstruction was performed (True Confocal Scanner, Leica Biosystems GmbH TCS SPE)."[26]
Jasra et al._2012	cardiac troponin-I (CT-I) and complement C9 (C9).	„ Formalin-fixed tissue samples were collected. Prepared paraffin blocks and H&E slides of all the cases were retrieved from the hospital records. All the paraffin blocks were cut into 4-μm sections for immunohistochemical staining with CT-I and C9. Sections for immunohistochemistry with CT-I and C9 were placed in an automated staining system. The prepared slides were analyzed under a light microscope. The cost of processing one sample ⁄tissue block was c. $40. Samples were considered positive for infarction when more than 10 adjacent cells were immunoreactive. Isolated myocytes reactivity was not considered as a positive classification of immunoreactivity. This was consistent with the widely held view that MI refers to infarctions of 1 cm or more across. Immunohistochemical Staining with Cardiac Troponin-I. The sections were deparaffinated and rehydrated. After blocking with iVIEW inhibitor, which is an endogen peroxidase, and preincubated for 4 min, sections were incubated with mouse anti-troponin-I monoclonal antibody diluted in 1:25 Dako antibody diluents for 24 min. The immunological reaction was visualized by diaminobenzidine (DAB) detection kit, which uses a peroxidase-conjugated avidin-biotin complex technique. The second layer was I-Biotin (Ventana Medical Systems), which was biotinylated goat anti-mouse IgG and IgM and biotinylated goat anti-rabbit IgG in phosphate buffer. Between each steps, the sections were washed with Tris buffer, pH 7.4. The color was developed using DAB, and the sections were counterstained with Harris hematoxylin. Immunohistochemical Staining with Complement 9. The

		sections were deparaffinated and rehydrated. After removal of the wax, the slides were washed with antigen retrieval. The sections were then blocked with iVIEW inhibitor and preincubated for 4 min. Sections were then incubated with mouse monoclonal antibody to human C9 diluted in 1:10 Dako antibody diluents for 24 min. An amplification kit was also added to the slides and was incubated for 8 min. The immunological reaction was visualized by DAB detection kit, which is using a peroxidase-conjugated avidinbiotin complex technique. The second layer was I-Biotin, which was biotinylated goat anti-mouse IgG and IgM and biotinylated goat anti-rabbit IgG in phosphate buffer. Between each steps, the sections were washed with Tris buffer, pH 7.4. The color was developed using DAB, and the sections were counterstained with Harris hematoxylin."[25]
Mathey et al._1994	C5b-9	„Myocardial tissue samples were quickly frozen in n-hexane cooled to — 6 °C by a mixture of dry ice in 100% ethanol and were kept at -196*C. Frozen sections (7 μm thick) were fixed for 5 min in cooled (4 *C) acetone and incubated for 2 h in a 1:40 dilution of polyclonal antibodies against C5b-9 complex. These antibodies detect antigenic sites in the C5b-9 molecule not present in the single complement factors composing the complex. The polyclonal antibodies were raised in a sheep by immunization with purified rabbit C5b-9 membrane complexes . They were subsequently purified from the sera by a membrane-elution procedure. The specificity of the purified IgG antibodies was verified by demonstrating lack of activity against native rabbit sera proteins in double-diffusion. The purified antibodies precipitated purified rabbit C5b-9 (m) and were also used as second antibodies to develop the C5b-9 (m) ELISA (see below). The preparations contained 0,2- 0,3 mg . IgG . ml^-1. After rinsing in phosphate-buffered saline, the sections were treated with the second antibody (anti-sheep IgG) by employing a kit (Vectastain) using a biotin-avidin (ABC) bridging technique and peroxidase as a marker enzyme. Peroxidase activity was stained by 3,3'-diaminopenzidine. All processing steps were done according to the instructions of the Vectastain kit. The histological sections were evaluated by two independent investigators who had no knowledge of the duration of coronary artery occlusion and the presence or absence of reperfusion according to the following criteria: 0=no C5b-9 deposition; +=single cell necrosis with staining of the cell membrane and rare intracellular granular staining; ++=staining of medium intensity of several myocytes; +++/; ++++=intense/very intense staining of the myocytes in large areas of infarction. The inter-observer variation applying this histological score was less than 5%."[17]

Sumitra et al._2005	cTnT, CK-MB mass and hsCRP	„30 μm sections were cut on a freezing microtome. To reduce endogenous peroxidase activity, free-floating sections were treated for 30 min with 0.3% H2O2 solution in 0.01 mol/L phosphate buffered saline (pH 7.4) containing 0.3% triton-X 100. These sections were incubated overnight at 37 ◦C with primary antibodies. The primary antibodies and the dilutions used were as follows: goat-antiC5 (Quidel, 1:50,000); goat anti-C6 (Calbiochem, 1:50,000); goat antiC7 (Quidel, 1:10,000); goat anti-C8 (Calbiochem, 1:50,000); goat anti-C9 (Quidel, 1:50,000) and sC5b-9 complex (Calbiochem, 1:50,000). The sections were then washed and treated with appropriate horseradish peroxidase conjugated IgG secondary antibodies for 2 h at 37 ◦C. Peroxidase labeling was visualized by incubation of the sections in 0.01% 3,3 -diaminobenzidine containing 0.6% nickel ammonium sulfate and 0.00015% H2O2 in 0.05% mol/L Tris–HCl buffer (pH 7.6). After colour development, sections were washed, mounted on glass slides, cover slipped with a gelatin solution. Controls were performed by omitting the primary antibody."[21]
Vakeva et al._1994	Complement C 1,3,8,9; CD59	„Hearts were removed immediately after killing of the animals. Tissue blocks were prepared for suitable horizontal tissue sections. One section was used for NBT staining and other parallel sections were placed in Tissue-Tek embedding medium (Ames Co., Elkhardt, IN), frozen using dry ice and stored at -70 C. Frozen sections (5 p) were fixed with cold acetone (10 minutes, -20 C) for indirect IFL microscopy. Serial sections were incubated for 30 minutes at 22 C with the following antibodies: mouse anti-rat CD59 mAb (TH9)26 or rabbit antibodies against rat Cl, C8, or C9.30 After washing three times with phosphatebuffered saline (PBS), pH 7.4, the sections were treated with fluorescein isothiocyanate (FITC-)- conjugated antibodies against rabbit or mouse (Dakopatts, Glostrup, Denmark) immunoglobulins, respectively. For detection of C3 FITC-conjugated antibodies against rat C3 (Cappel Laboratories, Malvern, PA) were used. IFL staining analyses were controlled by omitting the primary antibody, by using nonimmune sera, or by using mouse antibodies against rat albumin or rat IgG (Dakopatts). The IFL slides were mounted with Mowiol31 and examined on a Zeiss Standard microscope equipped with a filter specific for FITC fluorescence. For photography, Kodak Tri-X Pan films were used."[16]
Meng et al._2005	heart fatty acid binding protein (H-FABP)	„The heart was dissected and before it was fixed in 10% formalin in PBS over 24 h, a block under the ligation point was taken and fixed in 3.5% glutaraldehyde for electro-microscopic observation The tissue blocks for immunohistochemical stains and hemoxylin-eosin (HE) stains were taken transversely through the center of the

		infarcted area and embedded in paraffin according to routine histological procedures. The streptavidin-peroxidase conjugated method (S-P) was used for immunohistochemical staining of H-FABP. Sections of 4 μm were deparaffinized and dehydrated. After repairing the antigen by microwave, sections were pretreated with 0.3% hydrogen peroxide to block endogenous peroxidase activity and with the normal rabbit serum albumin, avoiding non-specific protein absorption. The sections were then incubated in the first antibodies (monoclonal antibody against rat H-FABP) at 37 8C for 1.5 h. After washing, they were incubated with the secondary anti-rabbit immunoglobulin conjugated biotin for 12 min and with the third antibody-streptavidin conjugated horseradish peroxidase for 12 min. Finally, the chromogen diaminobenzidine was used as substrate and the sections were weakly counterstained with hematoxylin. Control sections were treated with PBS instead of the primary specific antibody."[23]
Díaz et al._2005	cardiac troponin C (cTnC) and cardiac troponin T (cTnT)	„For the histological and immunohistochemical study, we obtained samples from zones of the heart showing signs of necrosis or hemorrhage (when present), or from the upper third of the intraventricular wall and upper third of the free wall of the left ventricle (those areas where myocardial infarction would most likely be situated). Histological studies with hematoxylin-eosin (H&E) staining and Masson's trichrome staining in formalin-fixed paraffin sections were performed. Immunohistochemical techniques using streptavidinbiotin-peroxidase according to the modificated Hsu' methods (Hsu et al., 1981) were performed. As primary antibodies we used monoclonal antibodies against subunits C and T of troponin (NCL-TROPC and NCLTROPT, Novocastra Lab Ltd, UK), which react with human cardiac troponin C and human fast muscle troponin T. The monoclonal antibody for subunit I is only suitable for fresh specimens, and did not serve for the controls included in paraffin (as controls we used tissue of normal heart). Because the immunohistochemical study was performed in material fixed in formol and included in paraffin, it was necessary to increase the reactivity of the tissue by means of a pretreatment involving tripsin digestion at 37 °C for five minutes. The working dilution of monoclonal antibodies were 1:40 and 1:20, respectively. For the rest of the immunohistochemical study, we used the LASAB kit (DAKO diag. Barcelona, Spain)."[22]
